# The Path of an Industrial Powder Small-Molecule Crystallographer

**DOI:** 10.1063/4.0000809

**Published:** 2025-10-27

**Authors:** James A Kaduk

**Affiliations:** North Central College, Naperville, IL, USA

## Abstract

In this historical talk, I will begin with some of the early history of powder diffraction in the US and my relation to it. My industrial and (fairly- convoluted) external career intersected with a number of significant developments in hardware, software, and databases - so I will look back on it, with emphasis on the interpersonal relationships which made it possible. I will end with a summary of the current status of powder crystallography (solving and refining crystal structures using powder data, in contrast to the more-normal applications of powder diffraction), and its future outlook.

